# Learning to play badminton altered resting-state activity and functional connectivity of the cerebellar sub-regions in adults

**DOI:** 10.1371/journal.pone.0223234

**Published:** 2019-10-01

**Authors:** Mengling Shao, Huiyan Lin, Desheng Yin, Yongjie Li, Yifan Wang, Junpeng Ma, Jianzhong Yin, Hua Jin

**Affiliations:** 1 Key Research Base of Humanities and Social Sciences of the Ministry of Education, Center of Cooperative Innovation for Assessment and Promotion of National Mental Health, Academy of Psychology and Behavior, Tianjin Normal University, Tianjin, China; 2 Institute of Applied Psychology, School of Public Administration, Guangdong University of Finance, Guangzhou, China; 3 Department of Radiology, Tianjin First Center Hospital, Tianjin, China; University of Pennsylvania, UNITED STATES

## Abstract

Previous studies have shown that sport experts are different from novices in functions and structures of the cerebellar sub-regions and the functional connectivity (FC) associated with the cerebellum, suggesting the role of the cerebellum on motor skill learning (MSL). However, the manipulation of individuals with different motor skills fails to exclude the effects of innate talents. In addition, individuals with higher motor skills often start with the MSL in their young ages. It is still unclear whether the effects regarding the cerebellum would be shown at one’s adult age. The present study was to directly alter individuals’ motor skills to investigate whether MSL (taking learning to play badminton as an example) in adulthood influences resting-state activity in the cerebellum. To this end, young adults without ball training experience were recruited as participants and were assigned randomly into the experimental group and the control group. Participants in the experimental group were asked to attend a badminton training course for 12 weeks, while the control group did not regularly attend any ball sports during this period. Resting-state functional magnetic resonance imaging (fMRI) was recorded before and after the training. Results showed that compared to the control group, the experimental group had smaller amplitude of low-frequency fluctuation (ALFF) in right cerebellar hemispheric VI and left VIII after training. For the experimental group, right hemispheric VIII had a stronger FC with left hemispheric IV-V, cerebellar vermal IX, left middle cingulate gyrus and right hippocampus after training. Taken together, these findings suggested that MSL, at least learning to play badminton in adulthood, reduces resting-state activity in different sub-regions in the cerebellum but increases FC between sub-regions of the cerebellum as well as between sub-regions of the cerebellum and cerebral cortices (e.g., middle cingulate cortex and hippocampus).

## Introduction

The human cerebellum, hidden underneath the occipital and behind the brain stem, is thought to be a heterogeneous structure consisting of the vermis and two hemispheres, and it has been anatomically divided into many sub-regions (i.e., vermis: vermal I-X, hemisphere: hemispheric I-X) [1]. Previous studies have repeatedly found that the cerebellum plays an important role in motor learning and acquisition of new skills [2–6]. Recently, the results from task-state neuroimaging and intrinsic functional connectivity (FC) studies demonstrated that different sub-regions of cerebellum have different functions in motor learning or skill learning, e.g., hemispheric IV-V and VIII associated with sensorimotor processing [7–11]; hemispheric VI involved in motor coordination [12–13]; vermal VI-IX responsible for eye movement control [10,14–16].

Researchers found that motor skill learning (MSL) influences cerebellar structures and functions by observing the corresponding structures and functions of sport experts and novices [17–22]. Regarding cerebellar structures, studies on gray matter (GM) showed that the volumes of hemispheric I, VI and VIIb were larger for judo athletes compared to novices [17]. The concentration and volumes of hemispheric VIII and VI were found to be larger for badminton athletes as compared to novices [18]. Studies on white matter (WM) showed that volumes of vermal VI-VII were larger for basketball athletes compared to novices [19–20]. For task-related cerebellar functions, the changes of blood oxygenation level-dependent (BOLD) signal in the hemispheric VI were found to be greater for professional archers [21] and hemispheric crus I (a part of hemispheric VII) for high jumpers [22] compared to novices, when participants imagined expert-related skills. In terms of resting-state cerebellar functions, Di et al. observed that badminton athletes had stronger amplitude of low-frequency fluctuation (ALFF) in vermal VI than novices [18].

With regards to FC, three studies found that MSL altered the FC among different sub-regions of the cerebellum and between cerebellum and other cerebral cortices [18, 23–24]. A resting-state fMRI study by Di et al. found that the cerebellum exhibited less FC with the right anterior cingulate cortex for athletes compared to novices [18]. Wang et al. showed that gymnasts as compared to novices had lower inter-modular among cerebellum, cingulo-opercular and frontal-parietal networks [23]. On the contrary, Kim et al. found that professional golfers had greater FC between the left cerebellum and cerebral cortices (e.g., occipital lobe, temporal gyrus, parietal lobes and frontal lobes) than novices [24]. These findings may suggest that MSL alter the function integration between cerebellum and cerebral cortices. The inconsistent results regarding cerebellar FC may be related to different types of sport events and differential sport experiences between experimental and control groups. The sport event was badminton in Di and colleagues, gymnastics in Wang and colleagues and golf in Kim and colleagues. Regarding sport experiences, the experimental group in Di and colleagues received an average of 8.9 years of professional badminton training. In the study of Wang and colleagues, participants in the experimental group received an average of 16.1 years of professional gymnastics training. In the study of Kim and colleagues, participants in the experimental group were professional golfers and had a training experience of 6 to 12 years. Previous studies have found the FC varies nonlinearly with the time of motor skill learning [25]. So, the differences in sport event and sport experience may alter cerebellar FC.

Notably, all of the aforementioned studies adopted cross-sectional design. This design cannot exclude the influence of innate factors on the effects of MSL. For instance, compared to novices, athletes are often thought to have higher talents in sports [26–28]. The athletes may have been born with specific cerebellar structures and functions that would confound with the effects of MSL. In addition, athletes often start with their trainings in their childhood. It has been demonstrated that brain plasticity is larger for children and adolescents compared to adults [29]. Therefore, it is still unclear whether MSL in the adulthood can alter structures, functions and FC associated with the cerebellum.

The present study aimed to use a longitude design to investigate the effects of MSL (taking learning to play badminton as an example) on the cerebellum in adults. To address this issue, young adults, who have no any ball training experience, were recruited as participants. They were assigned randomly to two different groups, i.e., the experimental group and the control group. Participants in the experimental group were asked to attend to a badminton training course (i.e., learning to play badminton) for 12 weeks, whereas for the control group, participants remained in their common life. Both of the two groups were scanned by fMRI before and after the training. Moreover, cross-sectional studies have suggested that professional ball training including badminton training alters cerebellar structures and functions [18–20]. Compared to other racquet sports, such as golf, it is cheaper and easier for badminton learning. Therefore, we would like to choose badminton as the training item.

The present study was interested only in the effects of MSL on resting-state functions and FC associated with the cerebellum. We would use the ALFF analysis to examine the regional properties of the intrinsic neural activity in the cerebellar sub-regions. The voxel-wise FC analysis was then conducted to characterize functional integration. Based on the literatures [7–16], the cerebellar sub-regions associated with sensorimotor processing (i.e., hemispheric IV-V and VIII), motor coordination (i.e., hemispheric VI) and eye movement (i.e., vermal VI-IX) were defined as regions of interest (ROIs) used to perform ALFF analysis and voxel-wise FC.

Task-state fMRI studies found that continuous skill learning lead to decreased activity in cerebellum [30–32]. And, the ALFF of resting-state fMRI is thought to have the same underlying electrophysiological mechanism as the task-induced fMRI BOLD signal [33–34]. Taken together, we assumed that after training, the ALFF of cerebellar sub-regions associated with motor and cognition processing in learning to play badminton would decrease in the experimental group, but not in the control group. For the FC, previous studies have demonstrated that brain regions from the same functional network or co-activated in relevant tasks have highly synchronous in spontaneous low-frequency fluctuation [35–37]. Therefore, we predicted that after training, the FC among the brain regions responsible for motor learning or cognition processing involved in learning to play badminton may become stronger in the experimental group, but not in the control group.

## Methods

### Participants

Thirty-six undergraduate and postgraduate students (23–26 years, *M* ± *SD* = 24.22 ± 0.83 years, 15 males) were recruited from Tianjin Normal University in return of payments. Half of the participants were assigned to the experimental group (23–26 years, *M* ± *SD* = 24.39 ± 0.92 years, 8 males), and the other half to the control group (23–26 years, *M* ± *SD* = 24.06 ± 0.73 years, 7 males). These two groups did not differ in age (*p* > 0.1) and education. All participants were right-handed as determined by the Edinburgh Handedness Inventory [38]. The participants had normal or corrected-normal visual and reported no history of neurological or psychiatric disorders or brain injuries. The participants did not report any amateur or professional trainings of badminton or other relevant ball sports (e.g., tennis and basketball) before the experiment. They seldom played badminton or other relevant ball sports or watched the matches or videos regarding related sports before the pre-test (less than once a month in the last three years). During the intervention, the experimental group participated in the 12-week badminton training. The control group kept their routine life (e.g., study and entertainments). However, they cannot attend any professional or amateur trainings of badminton or other relevant ball sports, play related sports, or watch related matches or videos. Informed consent was obtained from each participant prior to each test of the experiment. This study was approved by the local ethical committee (i.e., the ethical committee of Tianjin Normal University and the ethical committee of Tianjin First Center Hospital).

### Experimental procedures

Both the experimental and the control groups of participants were asked to attend two resting-state scans, once before the training and once after. The first scan was taken 17–10 days before the training, and the second scan was taken 1–5 days after the training.

The training lasted for 12 consecutive weeks, with 1 hour/time × 3 times for each week. The reason for the 12 weeks’ intervention was that previous studies have found that 12 weeks of training can trigger function changes in the concerned brain regions [39, 40]. Second, a semester includes 16–18 weeks. Considering that undergraduates are usually busy in social activities during the first few weeks of a semester and in exam during the last two weeks, a training program of 12-week in the mid of a semester is relatively easy to persuade participants to complete the whole experiment. The content of training is to learn playing badminton. Each time of training included three parts. In the first part, a teacher explained about badminton skills (e.g., serve, smash, anticipate the trajectory according to opponent’s body movements and fight the ball back to the left or the right court according to the position of opponent). In the second part, participants were asked to practice playing badminton in pairs, during which the teacher corrected the wrong movements or postures. In the last part, participants were asked to play badminton with their pairs freely. These three parts lasted for approximately 10, 30 and 20 minutes, respectively. In the last three times of the training, the teacher organized a badminton match. During the training, participants in the control group did not play with any ball games.

### MRI acquisition

The participants were scanned by a Siemens 3T Trio scanner with a standard 32-channel head coil in Tianjin First Center Hospital. The functional (T2-weighted) images were acquired using an echo-planar imaging (EPI) sequence. Acquisition parameters were as follows: TR = 2000ms, TE = 30ms, field of view = 220mm × 220mm, flip angle = 90°, matrix = 58 × 64, 25 slices/volume, thickness = 5mm, gap = 0.75mm, voxel size = 3.8mm × 3.4mm × 5mm. The scanning direction is from top to bottom. The scanning mode is interval scan, starting from the odd layer. The resting scan lasted 8min 26s for each participant. The structural (T1-weighted) images were acquired using gradient echo sequence. Acquisition parameters were as follows: TR = 1900ms, TE = 2.52ms, field of view = 240mm × 256mm, flip angle = 9°, matrix = 240 × 256, 176 slices, thickness = 1mm, gap = 0.5mm, voxel size = 1mm × 1mm × 1mm. The scanning direction is from right to left. The scanning mode is sequential scan. The T1 imaging scan lasted 4min 10s for each participant. In the scanner, participants were asked to keep awake with their eyes closed, without any motions or thinking.

### Resting-state fMRI data preprocessing

The preprocessing of resting-state fMRI data were performed using a toolbox for data processing and analysis of brain imaging, DPABI V2.1 (Copyright(c) 2014; GNU GENERAL PUBLIC LICENSE; http://rfmri.org/dpabi) [41], implemented in Matlab R2012a (MathWorks, http://www.mathworks.com/). For each participant, raw data were converted into NIFTI format, and the first ten-time points of resting-state fMRI data were discarded, leaving 240 time-points for further analysis. The further preprocessing included slice timing, head-motion correction (the data of those whose head motion > 1.5 mm in any plane and rotation > 1.5° in any direction were discarded), nuisance covariates regression (including polynomial trend, Fristion24, WM, and cerebrospinal fluid (CSF)), spatial normalization by information from unified segmentation (For each participant, structural image was first coregistered to the mean functional image. Then, the transformed structural image was segmented into GM, WM and CSF by using a unified segmentation algorithm. Next, functional images were normalized to the standard Montreal Neurological Institute (MNI) space using the normalization parameter estimated during unified segmentation.), spatial smoothing (full width at half maximum (FWHM) = 8 mm × 8 mm × 8 mm Gaussian kernel), and a band-pass filter within 0.01–0.08 Hz.

### ALFF analyses

**Fig 1:**
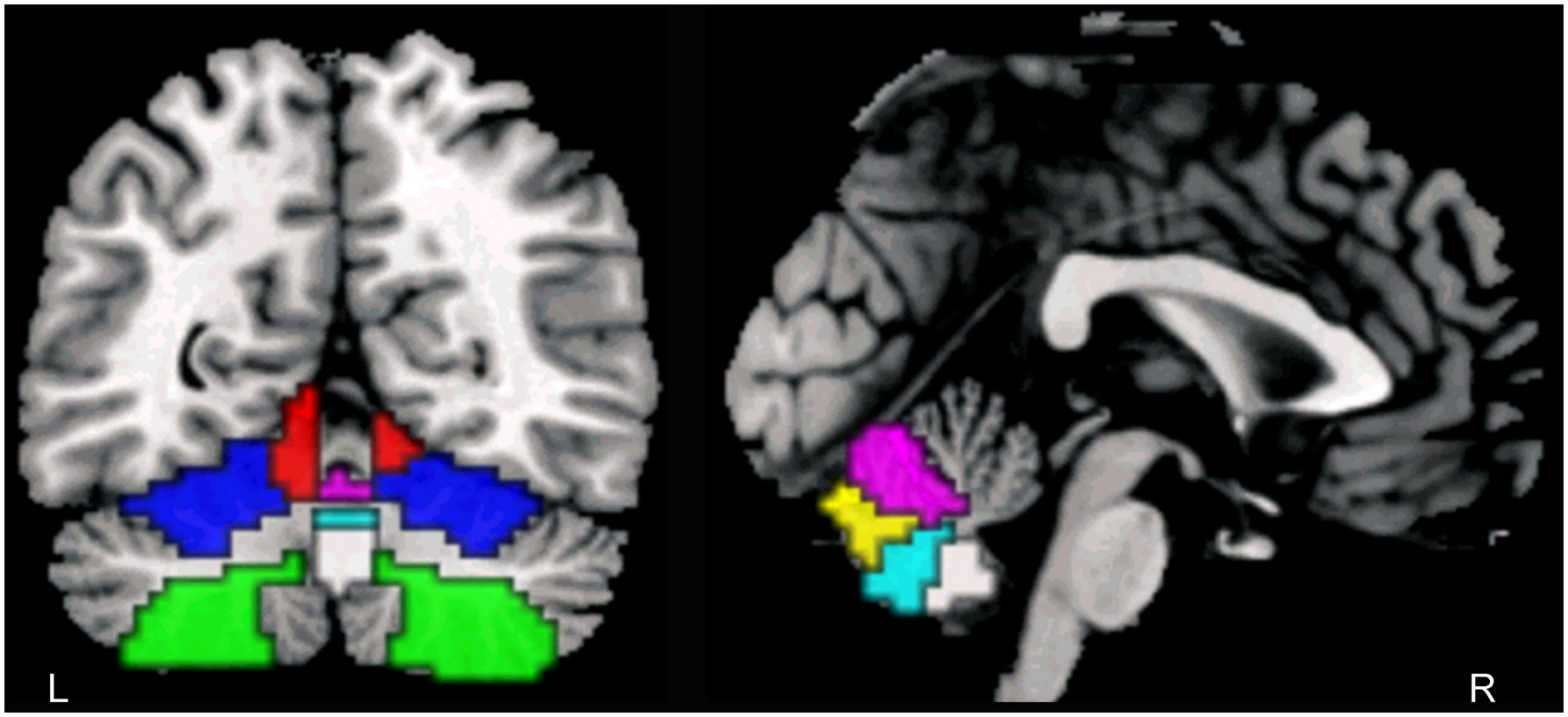
The locations of ROIs. Note: L: left; R: right; red: AAL97/98, cerebellar hemispheric IV-V; blue: AAL99/100, cerebellar hemispheric VI; green: AAL103/104, cerebellar hemispheric VIII; purple: AAL112, cerebellar vermal VI; yellow: AAL113, cerebellar vermal VII; cyan: AAL114, cerebellar vermal VIII; white: AAL115, cerebellar vermal IX.

The filtered data were used to perform ALFF analysis in DPABI V2.1. An ALFF map was calculated for each participant and a normalized ALFF map (mALFF) was also obtained for subsequent statistical analysis. According to the automated anatomical labeling (AAL) template [42], bilateral hemispheric IV-V, VIII, VI and vermal VI, VII, VIII and IX were defined as ROIs (The index and names of ROIs see Table 1, the locations of ROIs see Fig 1).

**Table 1:** The index and corresponding sub-regions of cerebellum from automated anatomical labeling (AAL) template.

Index	Region	Hemisphere
AAL91	Cerebellar hemispheric crus I	L
AAL92	Cerebellar hemispheric crus I	R
AAL93	Cerebellar hemispheric crus II	L
AAL94	Cerebellar hemispheric crus II	R
AAL95	Cerebellar hemispheric III	L
AAL96	Cerebellar hemispheric III	R
AAL97	Cerebellar hemispheric IV-V	L
AAL98	Cerebellar hemispheric IV-V	R
AAL99	Cerebellar hemispheric VI	L
AAL100	Cerebellar hemispheric VI	R
AAL101	Cerebellar hemispheric VIIb	L
AAL102	Cerebellar hemispheric VIIb	R
AAL103	Cerebellar hemispheric VIII	L
AAL104	Cerebellar hemispheric VIII	R
AAL105	Cerebellar hemispheric IX	L
AAL106	Cerebellar hemispheric IX	R
AAL107	Cerebellar hemispheric X	L
AAL108	Cerebellar hemispheric X	R
AAL109	Cerebellar vermal I and II	-
AAL110	Cerebellar vermal III	-
AAL111	Cerebellar vermal IV-V	-
AAL112	Cerebellar vermal VI	-
AAL113	Cerebellar vermal VII	-
AAL114	Cerebellar vermal VIII	-
AAL115	Cerebellar vermal IX	-
AAL116	Cerebellar vermal X	-

Note: AAL91-AAL108 belong to cerebellar hemispheric lobes. AAL109-AAL116 belong to cerebellar vermal lobes. AAL97-AAL100, AAL103-AAL104 and AAL112-AAL115 were the ROIs used for ALFF and FC analyses in the present study.

At group-level analysis, a full factorial design was carried out with a focus on the interaction of test time (pre vs. post) × group (the experimental vs. the control group) within each ROI in SPM8 (http://www.fil.ion.ucl.ac.uk/spm/) implemented in Matlab R2012a. Test time is a within-subject factor and group is a between-subject factor. The significant threshold was set voxel-level corrected *p*_FWE_ < 0.05 by Family-Wise Error and cluster size > 10 voxels. For each ROI, the ALFF signals were extracted, if the interaction effect was significant. Notably, the signals were based on the ROIs from the AAL template. Then, the signals were used to perform post-hoc *t*-tests with IBM SPSS Statistics software (Version 22; SPSS Inc., an IBM company, Chicago, Illinois). A probability level of *p* < 0.05 was considered statistically significant.

### FC analyses

The filtered data were used to perform whole-brain level FC analysis in DPABI V2.1. First, ten ROIs from AAL, which are the same as those in ALFF analyses, were selected as seeds. Subsequently, the average BOLD time courses from each ROI were extracted. The Pearson’s correlation coefficients between this average time course and the BOLD time course of every other voxel were computed. Then, the correlation coefficients were converted to Z-values (zFC) using Fisher transformation for subsequent statistical analyses.

At group-level analysis, a full factorial design was carried out with a focus on the interaction of test time (pre vs. post) × group (the experimental vs. the control group) in SPM8 implemented in Matlab R2012a. Test time is a within-subject factor and group is a between-subject factor. The significant threshold was set voxel-level corrected *p*_FWE_ < 0.05 by Family-Wise Error and cluster size > 10 voxels. For the zFC between each seed region and each of the other brain regions, the signals were extracted, if the interaction effect was significant. Notably, the signals of the seed regions were based on the ROI from the AAL template and the signals of the other brain regions were based on the voxel-level whole brain. Then, the signals were used to perform post-hoc analyses with SPSS 22.0 and the significant threshold level was *p* < 0.05.

## Results

### Results of ALFF

**Fig 2:**
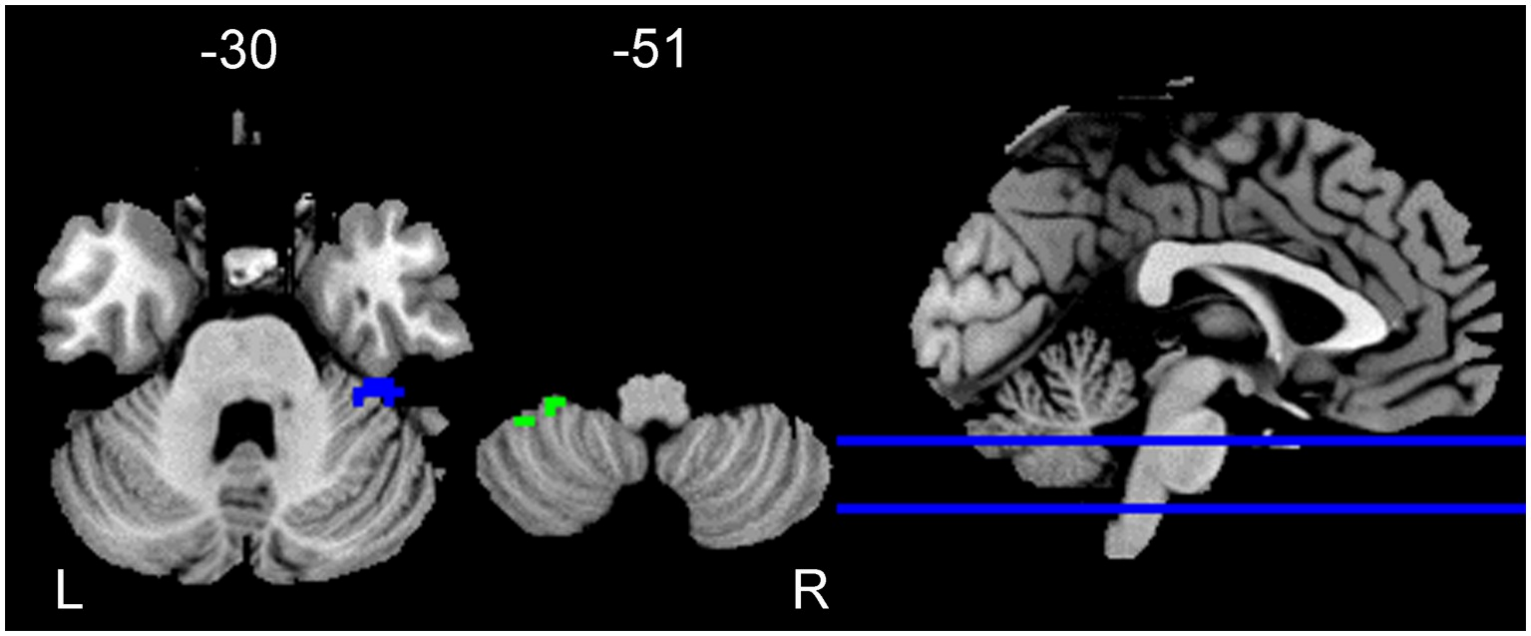
Group by test time interaction for the ALFF in cerebellar sub-regions. Threshold was set voxel-level corrected *p*_FWE_ < 0.05 by Family-Wise Error and cluster size > 10 voxels. Note: L: left; R: right; blue: right cerebellar hemispheric VI; green: left cerebellar hemispheric VIII.

A significant interaction between test time and group was found in the right cerebellar hemispheric VI and left cerebellar hemispheric VIII. No significant interaction was found in any other ROIs. See Table 2 and Fig 2.

**Table 2:** A significant interaction between group and test time in cerebellar ALFF.

ROI	voxels	*p*_FWE_	Peak *t*	*MNI* coordinates (mm)		
				*x*	*y*	*z*
R. cerebellar hemispheric VI	28	< 0.001	8.29	45	-33	-30
L. cerebellar hemispheric VIII	19	< 0.001	6.33	-30	-36	-51
		< 0.001	5.48	-42	-42	-45

Note: Threshold was set voxel-level corrected *p*_FWE_ < 0.05 by Family-Wise Error and cluster size > 10 voxels. L: left; R: right; MNI: Montreal Neurological Institute. The ALFF of right cerebellar hemispheric VI and left cerebellar hemispheric VIII had significant interactions between group and test time.

Regarding the ALFF of right cerebellar hemispheric VI, further analysis showed that for the experimental group, the ALFF was slightly smaller at post-test than at pre-test (*p* = 0.088). For the control group, there were no significant differences between pre-test and post-test (*p* > 0.1). The other direction of further analysis showed that at pre-test, there were no significant differences between the two groups (*p* > 0.1). At post-test, the ALFF was slightly smaller for the experimental group than for the control group (*p* = 0.078).

For the ALFF of left cerebellar hemispheric VIII, further analysis showed that there were no significant differences between pre-test and post-test in either the experimental group or the control group (*ps*. > 0.1). The other direction of further analysis showed that at pre-test, there were no significant differences between the experimental group and the control group (*p* > 0.1). At post-test, the experimental group had smaller ALFF than the control group (*p* = 0.005). See Table 3 in more details.

**Table 3:** The ALFF of the two groups at pre- and post-test in brain areas showing significant interaction.

ROI	group	pre-test (*M*±*SD*)	pos-test (*M*±*SD*)
R. cerebellar hemispheric VI	control	0.71±0.15	0.74±0.16
	experimental	0.73±0.11	0.66±0.11
L. cerebellar hemispheric VIII	control	0.44±0.07	0.46±0.08
	experimental	0.42±0.08	0.39±0.06

Note: L: left; R: right.

### Results of FC

**Fig 3:**
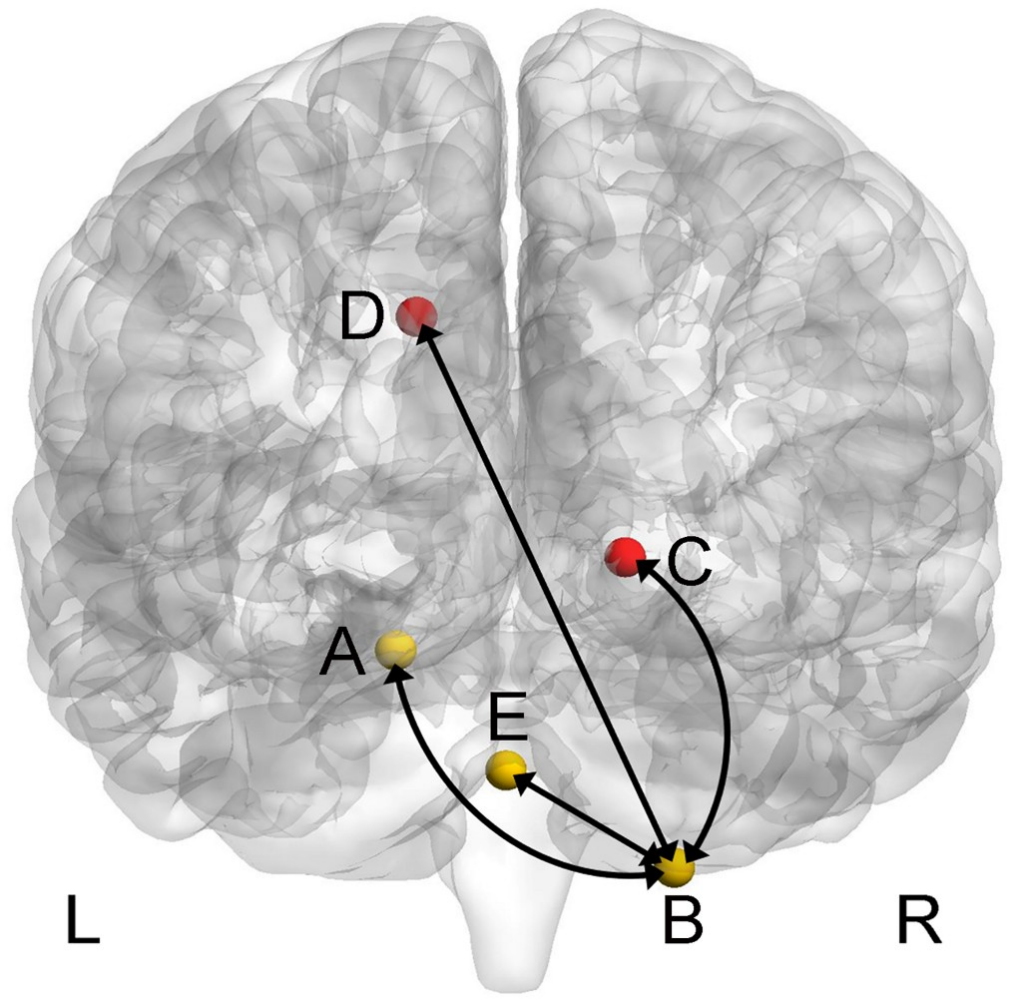
A significant interaction of FC among cerebellar sub-regions and cerebral cortexes. Threshold was set voxel-level corrected *p*_FWE_ < 0.05 by Family-Wise Error and cluster size > 10 voxels. Note: L: left; R: right; yellow: cerebellar sub-regions; red: cerebral cortexes. The experimental group had stronger FC between left cerebellar hemispheric IV-V (A) and right cerebellar hemispheric VIII (B); between right cerebellar hemispheric VIII (B) and right hippocampus (C); between right cerebellar hemispheric VIII (B) and left middle cingulate gyrus (D); between cerebellar vermal IX (E) and right cerebellar hemispheric VIII (B) after training. However, these FC of the control group decreased at post-test compared to pre-test.

**Fig 4:**
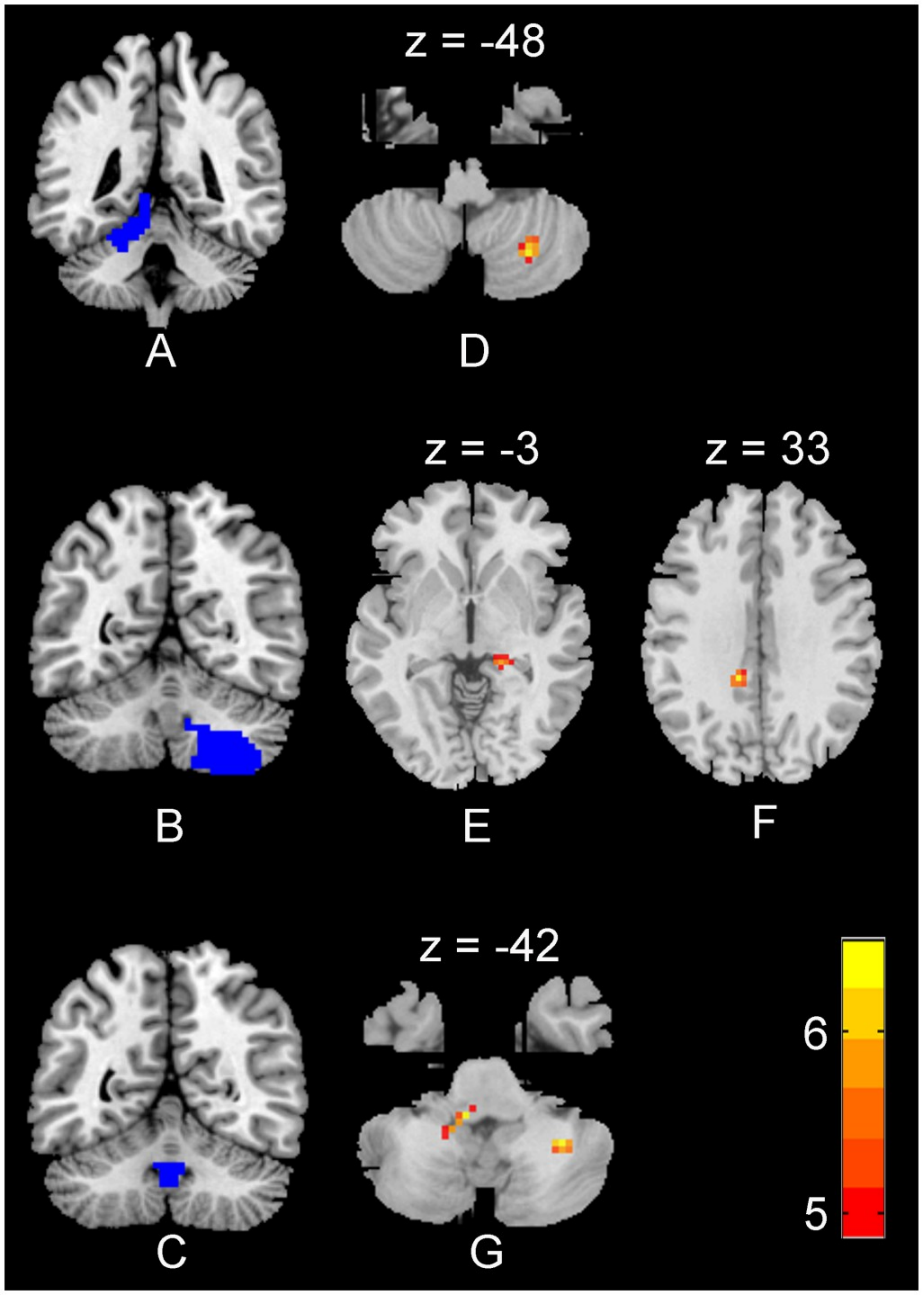
A SPM results map revealing a significant interaction of FC among cerebellar sub-regions and cerebral cortexes. Threshold was set voxel-level corrected *p*_FWE_ < 0.05 by Family-Wise Error and cluster size > 10 voxels. There was a significant interaction in FC between left cerebellar hemispheric IV-V (A) and right cerebellar hemispheric VIII (D); between right cerebellar hemispheric VIII (B) and right hippocampus (E); between right cerebellar hemispheric VIII (B) and left middle cingulate gyrus (F); between cerebellar vermal IX (C) and right cerebellar hemispheric VIII (G).

A significant interaction between test time and group was found between left cerebellar hemispheric IV-V and right cerebellar hemispheric VIII, between right cerebellar hemispheric IV-V and right brainstem pons, between right cerebellar hemispheric VIII and right hippocampus, between right cerebellar hemispheric VIII and left middle cingulate gyrus, between vermal IX and right cerebellar hemispheric VIII and between vermal IX and left brainstem pons. No significant interaction was found in any other ROIs. See Table 4, Figs 3 and 4 in more details.

**Table 4:** A significant interaction between group and test time in FC.

ROI	Area	voxels	*p*_FWE_	Peak *t*	*MNI* coordinates (mm)		
					*x*	*y*	*z*
L. hemispheric IV-V	R. cerebellar hemispheric VIII	29	0.003	5.80	27	-63	-48
R. hemispheric IV-V	R. brainstem pons	10	0.001	6.03	6	-21	-24
R. hemispheric VIII	R. hippocampus	16	0.002	5.79	18	-33	-3
	L. middle cingulate gyrus	15	< 0.001	6.51	-12	-39	33
Vermal IX	R. cerebellar hemispheric VIII	13	0.011	5.36	33	-51	-42
	L. brainstem pons	11	0.011	5.34	-9	-39	-42

Note: Threshold was set voxel-level corrected *p*_FWE_ < 0.05 by Family-Wise Error and cluster size > 10 voxels. L: left; R: right; MNI: Montreal Neurological Institute. The FC among these brain regions had significant interactions.

Further analysis showed that for the experiment group, the FCs in each of abovementioned two brain areas were larger in the post-test than in the pre-test (*ps* < 0.05). However, for the control group, those FCs were smaller in the post-test than in the pre-test (*ps* < 0.05).

The other direction of the further analysis showed that at the pre-test, the experimental group had smaller FC in in each of abovementioned two brain areas than the control group (*ps* < 0.05). However, at the post-test, the effect regarding FC was reserved (*ps* < 0.05). See Table 5 in more details.

**Table 5:** The FC of the two groups at pre- and post-test.

Brain areas	group	pre-test (*M*±*SD*)	post-test (*M*±*SD*)
between L. cerebellar hemispheric IV-V and R. cerebellar hemispheric VIII	control	0.33±0.18	0.12±0.18
	experimental	0.10±0.12	0.26±0.11
between R. cerebellar hemispheric IV-V and R. brainstem pons	control	0.51±0.19	0.27±0.21
	experimental	0.29±0.15	0.46±0.16
between R. cerebellar hemispheric VIII and R. hippocampus	control	0.44±0.20	0.22±0.21
	experimental	0.26±0.17	0.45±0.11
between R. cerebellar hemispheric VIII and L. middle cingulate gyrus	control	0.37±0.12	0.19±0.17
	experimental	0.14±0.07	0.27±0.12
between cerebellar vermal IX and R. cerebellar hemispheric VIII	control	0.55±0.25	0.22±0.23
	experimental	0.32±0.19	0.47±0.15
between cerebellar vermal IX and R. cerebellar hemispheric VIII	control	0.69±0.21	0.45±0.16
	experimental	0.49±0.15	0.65±0.12

Note: L: left; R: right.

Given that the group effect regarding the abovementioned FCs in the post-test might be due to the group effect in the pre-test as a result of significant pre-test group effect, we performed ANCOVAs on each post-test FC of each of the abovementioned two regions using pre-test FCs as a covariate. Results showed that the post-test FC was larger for the experimental group than the control group (left cerebellar hemispheric IV-V and right cerebellar hemispheric VIII: 0.30±0.04 vs. 0.07±0.04 (experimental vs. control); right cerebellar hemispheric IV-V and right brainstem pons: 0.54±0.04 vs. 0.20±0.04; right cerebellar hemispheric VIII and right hippocampus: 0.48±0.04 vs. 0.19±0.04; right cerebellar hemispheric VIII and left middle cingulate gyrus: 0.34±0.04 vs. 0.12±0.04; vermal IX and right cerebellar hemispheric VIII: 0.47±0.05 vs. 0.23±0.05; vermal IX and left brainstem pons: 0.69±0.03 vs. 0.41±0.03, *ps* < 0.05). Therefore, the results indicate that badminton training is effective on influencing FCs, even though the FCs were different in experimental and control groups before the training.

## Discussion

The present study aimed to investigate whether motor skill learning, taking learning to play badminton as an example, in adulthood influenced resting-state activity of hemispheric IV-VI, VIII and vermal VI-IX of the cerebellum and intra-modular FC within cerebellum and inter-modular between cerebellum and cerebral cortices. The results showed decreased ALFF activity in right hemispheric VI and left hemispheric VIII of the cerebellum for the experimental group but not for the control group after training, suggesting the role of learning to play badminton on the resting-state activity of the cerebellum. In addition, the experimental but not the control group exhibited stronger FC between the several sub-regions of cerebellum and some other regions of cerebral cortices or cerebellum after training, e.g., between left cerebellar hemispheric IV-V and right cerebellar hemispheric VIII, between right cerebellar hemispheric VIII and left middle cingulate gyrus, between right cerebellar hemispheric VIII and right hippocampus, between cerebellar vermal IX and right cerebellar hemispheric VIII. These findings suggest that MSL integrates motor and cognition functions between different sub-regions of the cerebellum and between cerebellar sub-regions and cerebral cortices.

### The effects of learning to play badminton on the ALFF of the cerebellum

Previous studies showed that continuous skill learning affected the function of the cerebellum [30–32]. For example, in the study by Sacco et al. [31], participants attended a tango training session every day for 5 consecutive days and underwent task-state fMRI scans before and after the training. In the scanner, participants were asked to close their eyes and imagine they were walking along two parallel lines. ALFF is thought to reflect the regional properties of the brain’s spontaneous neural activity [34]. And the ALFF of resting-state fMRI is sought to have the same underlying electrophysiological mechanism as the task-induced fMRI BOLD signal [33, 34]. Therefore, in line with those literatures, the reduced ALFF in the sub-regions of cerebellum (right hemispheric VI and left hemispheric VIII) in the experimental group (compared to the control group) observed in our study may attribute to reduced activations of these sub-regions along with the skill learning of playing badminton.

As mentioned in the introduction, hemispheric VIII is related to sensorimotor [7–10]. This region was found to be activated during certain movements [7,8]. For example, in Grodd et al.’s study [7], participants were asked to perform several movement tasks with their eyes closed. They found that flexing or extending the foot activated hemispheric IV; flexing or extending hand activated hemispheric V and VIII; flexing or extending both elbow and wrist activated hemispheric V; tapping right fingers activated hemispheric V and VIII. Stoodley et al. also found that right-handed finger-tapping activated right hemispheric VIII, whereas this region was not activated in cognitive tasks that were unrelated to certain movements (e.g., language, working memory and mental rotation) [8].

The hemispheric VI in the cerebellum has been demonstrated to be involved in motor coordination in complex movements, such as visually-guided movements [13] and sequential movements [12]. For example, in the study by Alahmadi et al. [13], bilateral cerebellar hemispheric VI was activated, when participants were asked to complete a complex grip task with their right hand according to a visual cue defining the target force. Schlerf et al. [12] asked participants to perform simple (i.e., simultaneous flexion and extension of many digits) and complex movement tasks (i.e., sequences of individual digit flexion and extension) with either their fingers or toes. The results showed that hemispheric VI in the cerebellum was activated only in the complex movement tasks rather than the simple tasks.

Compared with the above tasks, learning to play badminton in real life is more complicated and it involves various kinds of motor plan and execution and cognition processes, such as, the movements of the body and limbs, hand-eye coordination and body limb coordination. The cerebellar hemispheric VI and VIII might be activated during learning to play badminton. Therefore, learning to play badminton may repeatedly activate the aforementioned sub-regions of the cerebellum, and lead to their low activities in the resting state. The findings suggest that skill learning of playing badminton decreases the spontaneous neural activity in right hemispheric VI and left hemispheric VIII of the cerebellum.

Different from the findings in the present study, Di et al. found that badminton athletes had stronger ALFF in vermal VI than novices [18], however, the present study did not observe significant changes of ALFF in vermal VI. As mentioned above, vermal VI is related to eye movement control [10,14–16]. For athletes, the stronger ALFF in vermal VI may be due to their innate advantage of playing badminton.

### The effects of learning to play badminton on cerebellar-related FC

FC reflects the temporal correlation of low frequency fluctuations in the resting-state in the different brain regions [35, 43]. Brain regions from the same functional network or co-activated in relevant tasks have highly synchronous in spontaneous low-frequency fluctuation [35–37]. Therefore, the present study showed the experimental group had stronger FC between sub-regions of the cerebellum and between sub-regions of the cerebellum and cerebral cortices after badminton training, which may be related to their enhanced temporal synchronies, due to their co-activations during learning to play badminton. As mentioned above, cerebellar hemispheric VIII and hemispheric IV-V are two sensorimotor regions of cerebellum [7–11]. These regions were activated during overt movements [7–8]. Regarding the vermal IX of the cerebellum, this region is thought to be related to oculomotor movements, such as reflexive visually guided saccades and smooth pursuit [10, 14–15]. Badminton playing relies largely on dynamic visual information. For example, players track the trajectory of a shuttle and posture change of an opponent on the opposite side of a net. These processes require coordination of eye movements. Thus, co-activation for these sub-regions of the cerebellum is necessary and inevitable during badminton playing.

On the other hand, Badminton is set to play in a court with certain length and width. During badminton playing, to strike the ball back within the court, players have to intercept the shuttle from their opponents and strike the ball back with their racquets. Playing badminton involve not only motor plan and execution but also spatial perception and mental motor extrapolation (anticipation the future location of a moving shuttle). Right hippocampus is thought to be associated with the processing of spatial information, such as space navigation [44]. FMRI studies [45–48] found that when participants were anticipating upcoming outcomes of a moving object, left or right middle cingulate gyrus was activated for athletes compared to non-athletes. It is reasonable that these cerebral areas co-activate with sub-regions of cerebellum during badminton playing, leading to their stronger FC. Our findings indicate that skill learning of playing badminton not only increased intra-modular function integration within cerebellum but also inter-modular function integration between cerebellum and cerebral cortices.

## Limitations

We would like to mention limitation of our study and suggest outlines for future research. Firstly, as mentioned above, the duration of MSL may influence the effect of learning on the ALFF of the cerebellum. Future studies may extend the duration of learning to further investigate the related issue. Secondly, the present study did not exclude the influences of some factors, such as motor skills and experiences (e.g., computer games, instrument trainings and driving), physical characters (height and weight) and abilities of memory, learning, and emotional control. According to the results regarding the group effects, these factors or some of them might have led to differential FCs between experimental and control groups in the pre-test. While the ANCOVA analyses suggested the effectiveness of badminton training even though there were some pre-test differences between experimental and control groups, future studies should control for those factors and reduce the group differences in the pre-test to further investigate the related issues. Finally, it is not clear whether the results can generate in other badminton-similar sports (e.g., tennis) or even badminton-dissimilar sports (e.g., running). Future studies may include another control group, who attend the trainings of these badminton-similar and badminton-dissimilar sports, to further investigate the related issues.

## Conclusion

In the present study, we observed that compared to the control group, the experimental group had reduced the ALFF in right cerebellar hemispheric VI and left cerebellar hemispheric VIII after training. In addition, the experimental group exhibited stronger FC (e.g., between cerebellar hemispheric VIII and hemispheric IV-V, vermal IX, left middle cingulate gyrus and hippocampus.) after training. These findings indicate that these brain regions may be activated or co-activated during learning to play badminton, which leads to their lower ALFF and stronger FC at resting-state. The findings may contribute to understanding the changes of cerebellar plasticity induced by MSL, at least learning to play badminton in adults.

## Supporting information

S1 File The data regarding full factorial design for ALFF. (ZIP)

S2 File The data regarding full factorial design for FC. (ZIP)

S3 File The data used to perform post-hoc analysis. (ZIP)

S4 File The data regarding the results of post-hoc analysis. (ZIP)

S5 File The data regarding the results of ANCOVA. (ZIP)

S6 File The data regarding the ALFF of cerebral cortex. (ZIP)

S7 File The data regarding the FC between whole cerebellum and cerebral cortex. (ZIP)
